# MicroRNA-guided regulation of heat stress response in wheat

**DOI:** 10.1186/s12864-019-5799-6

**Published:** 2019-06-13

**Authors:** Sridhar Ravichandran, Raja Ragupathy, Tara Edwards, Michael Domaratzki, Sylvie Cloutier

**Affiliations:** 1 Agriculture and Agri-Food Canada, Ottawa Research and Development Centre, 960 Carling Avenue, Ottawa, Ontario K1A 0C6 Canada; 2 Plant Science Department, University of Manitoba, Winnipeg, Manitoba Canada; 3 Department of Computer Science, University of Manitoba, Winnipeg, Manitoba Canada; 4 Present address: Agriculture and Agri-Food Canada, Lethbridge Research and Development Centre, Lethbridge, Alberta Canada

**Keywords:** microRNA, Epigenetics, Heat stress, Wheat, Degradome, PARE, Post-transcription

## Abstract

**Background:**

With rising global temperature, understanding plants’ adaptation to heat stress has implications in plant breeding. MicroRNAs (miRNAs) are small, non-coding, regulatory RNAs guiding gene expression at the post-transcriptional level. In this study, small RNAs and the degradome (parallel analysis of RNA ends) of leaf tissues collected from control and heat-stressed wheat plants immediately at the end of the stress period and 1 and 4 days later were analysed.

**Results:**

Sequencing of 24 small RNA libraries produced 55.2 M reads while 404 M reads were obtained from the corresponding 24 PARE libraries. From these, 202 miRNAs were ascertained, of which mature miRNA evidence was obtained for 104 and 36 were found to be differentially expressed after heat stress. The PARE analysis identified 589 transcripts targeted by 84 of the ascertained miRNAs. PARE sequencing validated the targets of the conserved members of miRNA156, miR166 and miR393 families as squamosa promoter-binding-like, homeobox leucine-zipper and transport inhibitor responsive proteins, respectively. Heat stress responsive miRNA targeted superoxide dismutases and an array of homeobox leucine-zipper proteins, F-box proteins and protein kinases. Query of miRNA targets to interactome databases revealed a predominant association of stress responses such as signalling, antioxidant activity and ubiquitination to superoxide dismutases, F-box proteins, pentatricopeptide repeat-containing proteins and mitochondrial transcription termination factor-like proteins.

**Conclusion:**

The interlaced data set generated in this study identified and validated heat stress regulated miRNAs and their target genes associated with thermotolerance. Such accurate identification and validation of miRNAs and their target genes are essential to develop novel regulatory gene-based breeding strategies.

**Electronic supplementary material:**

The online version of this article (10.1186/s12864-019-5799-6) contains supplementary material, which is available to authorized users.

## Background

Rising global temperatures with increasing occurrences of heat waves negatively impact crop productivity and global food production [1]. Heat stress disrupts membranes and inactivates proteins by unfolding, misfolding and aggregation [2]. Such metabolic imbalances have an adverse impact on all aspects of plant growth and impair several physiological and developmental processes including photosynthesis, respiration, reproduction, grain filling and yield [reviewed by [3] ]. Plants perceive and respond to heat stress using an array of stress signaling pathways to regulate their metabolism and cell function. Perception of heat stress begins at the plasma membrane, with subsequent alterations in membrane fluidity, lipid saturation and protein stability [4]. Changes in membrane fluidity initiate Ca^2+^ influx and rapid accumulation of reactive oxygen species (ROS) such as H_2_O_2_ [4, 5]. Stress-induced Ca^2+^ influxes and ROS activate mitogen-activated protein kinase (MAPK) and calcium-dependent protein kinase (CDPK) stress signaling pathways [6, 7] to regulate the expression of heat stress transcription factors [8] and WRKY proteins in order to confer thermotolerance [9].

Small RNAs (sRNAs) are a class of endogenous non-coding RNAs that regulate gene expression at transcriptional and post-transcriptional levels. In plants, sRNAs are classified based on their biogenesis and function. MicroRNAs (miRNAs) are primarily 21 nucleotide (nt)-long molecules derived from single stranded precursors with a hairpin structure. miRNA coding genes are transcribed by RNA polymerase II (Pol II) to generate primary miRNAs (pri-miRNAs) that fold into characteristic self-complementary stem-loop secondary structures [10]. The pri-miRNAs are cleaved at least twice by Dicer-like1 (DCL1) endonucleases to yield the precursor-miRNAs (pre-miRNAs) that are further processed to release miRNA/miRNA* duplexes. Mature miRNAs are loaded onto Argonaute 1 (AGO1) to form miRNA-induced silencing complexes (miRISCs) that guide target-specific mRNA cleavage or translational repression [reviewed by [11] ]. In contrast, short interfering RNAs (siRNAs) are transcribed from transposons and repetitive regions by RNA polymerase IV (Pol IV). The single stranded RNA transcripts are copied into double-stranded RNA (dsRNA) molecules by RNA-dependent RNA polymerase 2 (RDR2) that are cleaved by DCL3 to produce the 24-nt siRNAs. These siRNAs are loaded onto AGO4 to induce sequence-specific transcriptional gene silencing (TGS) by recruiting methyltransferases [12].

miRNA-guided post-transcriptional gene silencing (PTGS) has emerged as a mechanism to reprogram the expression of development and stress associated genes. PTGS regulates nucleic acid binding [13, 14], phytohormone biosynthesis [15, 16], chlorophyll biosynthesis [17], flowering [18], root differentiation [19], nutrient homeostasis [20] and stress responses [21]. Earlier studies on Arabidopsis (*Arabidopsis thaliana*) and rice (*Oryza sativa*) identified a high degree of conservation among miRNAs and their mRNA targets [22]. While some miRNAs and their targets are evolutionarily conserved, many are lineage or even species-specific [11, 23]. The emergence of high-throughput sequencing technologies and genome-wide analysis has facilitated the identification of new miRNA variants from an ever increasing number of plant species [24, 25]. Similarly, computational approaches remain a valuable tool to predict plant miRNA targets by complementary base pairing in cDNA and whole genome sequences [22, 26]. However, experimental validation of miRNA-guided cleavage site is still required considering the high rate of false positive target prediction [27, 28]. Parallel analysis of RNA ends (PARE), also known as degradome analysis, has enabled the massive cloning and sequencing of 5′ ends of cleaved or uncapped mRNA to validate many miRNA-guided cleavage sites [29].

Advancements in sequencing technologies have revealed rapid divergence and high diversity in miRNAs and their targets [24, 25, 30]. Stress responsive miRNAs and their targets have been identified from several cereal crops including wheat, rice, maize and barley (reviewed in [31]). Despite the identification of miRNA from an increasing number of plant species and stress responses, experimental validation of miRNA targets is lacking in polyploid crops including wheat. However, studies in Arabidopsis and rice have experimentally validated a few miRNA targets regulating thermotolerence. For example, in Arabidopsis miR156-overexpressing plants exhibited enhanced tolerance to heat stress and was shown to be required for plant heat stress memory [32]. In Arbidopsis, plants expressing miR398-resistant version of *CSD* (copper/zinc superoxide dismutase) were more sensitive to heat stress. miR159 is downregulated in response to heat stress in order to regulate MYB-like family transcription factors. Transgenic rice plants overexpressing miR159 were more sensitive to heat stress [33].

In wheat, heat stress responsive miRNAs have been reported from several susceptible and resistant cultivars, primarily investigating early responses within 24 h of treatment and the role of isomiRs and differentially accumulated mature miRNAs among families was largely unnoticed [34–36]. Further, miRNA target identification in previous studies [34–37] have relied on prediction models based on draft genome assemblies. Although advancements in sequencing technologies have benefited the identification of several stress responsive miRNAs, lack of a high quality reference sequence and high confidence annotations have limited previous studies with the identification of miRNAs, isomiRs and validation of their targets.

Previous studies in cotton [38] and cowpea [39] have also shown genotype-specific miRNA expression profiles in responses to stress. In cereal crops such as rice, domestication has led to specific gain and/or loss of miRNA regulated gene expression [40]. As new miRNA variants are spawned and lost frequently, especially in polyploid crops such as wheat, accurate identification and experimental validation of miRNAs and their targets in response to heat stress is essential to develop novel regulatory-RNA-based plant breeding strategies. The recent release of the Chinese Spring wheat reference sequence [41] with high-confidence annotations has enabled the accurate identification of miRNAs and validation of their targets. The gold standard reference sequence can further be utilized to functionally validate miRNA targets.

We previously showed the temporal expression profile of wheat cv Glenlea with a pronounced differential expression of miRNA occurring immediately after heat stress until 3 days post treatment [37]. Here, wheat cv Chinese Spring was used to generate the interlaced data set of small RNA and PARE. The recently released wheat reference sequence [41] of cv Chinese Spring was used to enhance the accuracy of miRNA and isomiRs annotation and to validate heat stress regulated miRNAs target genes associated with thermotolerance. Heat stress regulated miRNAs were highly altered immediately after heat stress to regulate thermotolerance but expression levels largely returned to control levels during the 4 day recovery period following the end of the stress. Differential accumulation of isomiRs indicates highly regulated post transcriptional modifications dictating the accumulation of specific mature miRNAs in response to heat stress. Degradome sequencing revealed chloroplast and mitochondria localized pentatricopeptide repeat-containing protein and mitochondrial transcription termination factor-like protein targets that were previously not known to undergo miRNA-guided cleavage.

## Methods

### Plant materials and growth conditions

*Triticum aestivum* cv Chinese Spring seeds were obtained from Dr. Eric Kerber (Agriculture and Agri-Food Canada, Winnipeg, Canada). A single Chinese Spring plant was selfed to produce the seeds for this experiment. A total of 144 plants were grown in a PG-40 growth cabinet (Conviron Technologies, Winnipeg, Canada) under long-day conditions of 16 h of light (300 μmol m^− 2^ s^− 1^) at 18 °C and 8 h of darkness at 16 °C. At the boot stage, a set of 72 plants were exposed to a 37 °C heat stress for 5 days while the other 72 plants remained under the control conditions mentioned above. The heat stressed plants were amply watered twice per day during the 5-day stress period to avoid a confounding effect with drought stress. After the 5-day stress exposure, the plants were returned to the control conditions. The experiment was setup as a completely randomized design with four biological replicates, two treatments (control and heat) with 18 plants per treatment and replicate.

### Sampling and RNA extraction

Leaf tissue was collected from all heat-stressed and control individual plants immediately at the end of the stress period, i.e., time point zero day after treatment (0 DAT), and at one and four days after treatment (1 and 4 DAT). Leaf tissue from individual plants was immediately frozen in liquid nitrogen. Total RNA was isolated from all 432 leaf tissue samples using TRI-reagent (Ambion, Naugatuck, CT) as previously described [37]. Total RNA quality and quantity were obtained using an Agilent 2100 Bioanalyzer with the RNA 6000 Nano chip (Agilent Technologies, Santa Clara, CA). Equimolar amounts of RNA from the 18 individual leaf samples representing a replicate, treatment and time point were pooled to create the 24 total RNA samples representing the four biological replicates, two treatments and three time points. These 24 total RNA samples were used to construct sRNA and degradome libraries as described below.

### Small RNA library preparation, data processing and annotation of miRNA

sRNA libraries were constructed from low molecular weight (LMW) RNA isolated from 72 μg of total RNA as described by Lu and Souret (2010) [42]. Briefly, sRNA purified from LMW RNA was size-selected on a 15% polyacrylamide gel. A gel slice, corresponding to the 16 to 30 nt size range was eluted to obtain the sRNA fractions that were subsequently quantified on an Agilent 2100 Bioanalyzer with the Small RNA kit. The TruSeq® small RNA sequencing adapters (Illumina, San Diego, CA) were ligated to the size-selected sRNA samples and libraries were constructed using Illumina TruSeq® small RNA kit following the manufacturer’s instructions. The libraries were quantified on an Agilent 2100 Bioanalyzer using a High Sensitivity DNA kit. Six pools, each containing equimolar amounts of 12 random libraries and where each library was present in three different pools, were resolved on a 6% polyacrylamide gel from which the 145–160 bp fragments, corresponding to the adapter-ligated sRNA, were size-selected. The ethanol precipitated library pools were resuspended and quantified on an Agilent 2100 Bioanalyzer with the DNA 1000 kit. The library pools were normalized to 2 nM and sequenced on a MiSeq for 50 cycles using a MiSeq Reagent Kit v2 (Illumina). A total of six runs were performed to sequence the six pools where each library was sequenced in three separate runs.

A tool chain for processing small RNA reads [37] was used with slight modifications. miRNA annotation was carried out following the guidelines of [43] and A Kozomara and S Griffiths-Jones [44] for mature miRNA and precursor evidence, respectively. Distinct tags represented by ≥10 reads per million (RPM) in at least one library were mapped to the pre-miRNA loci identified from the hexaploid bread wheat genome (IWGSC RefSeq v1.0., 2018) using Bowtie2 [45]. Distinct tags with perfect matches were extracted for hairpin structure prediction using RNAfold [46]. Star sequences were predicted using a custom Python script.

Only the distinct tags that showed evidence of biosynthesis based on matching precursor that could fold into secondary hairpin structure with <− 0.2 kcal/mol/nt were retained. In addition, presence of the complementary star sequence on the opposite strand with a 2-bp offset on the 3′-end of the hairpin structure was assessed. Distinct tags represented by ≥10 RPM in at least one library were also matched to previously annotated miRNA and star sequences [47]. Distinct tags with strong evidence of biosynthesis, including precursor and star sequence supports, were selected for differential expression analysis and to identify miRNA target using degradome data.

Read counts were grouped to define treatment combination and normalized [48] to account for differences in library sequencing depth and RNA composition using edgeR [49]. MDS plots were constructed to check for outliers. After estimating dispersion a general linear model (GLM) was used with glmLRT function to identify miRNAs differentially expressed across treatments and over time. miRNAs with false discovery rate (FDR) < 0.05 [50] were considered differentially expressed. Hierarchical clustering ananlysis was performed using the hclust function in R. Heat maps were generated with normalized log2 read counts using the maximum algorithm and complete linkage method on ClustVis [51].

### Sequence extraction and multiple sequence alignment

The precursor sequences from model plants *Arabidopsis thaliana* (ath), *Brachypodium distachyon* (bdi) and cereal crops including *Oryza sativa* (osa)*, Sorghum bicolor* (sbi)*, Hordeum vulgare* (hvu) *and Zea mays* (zma) were extracted from miRBase release 22. Multiple sequence alignment was performed using MUSCLE with default parameters.

### Degradome

Degradome libraries were constructed from 75 μg of total RNA as previously described [52]. cDNA synthesized from 5′ adapter-ligated poly(A) RNA was digested with *Mme*I. After 3′ adapter ligation, double-stranded DNA (dsDNA) was purified on a gel and the libraries were PCR-amplified. The 128-bp fragment was size-selected and verified on an Agilent 2100 Bioanalyzer using a High Sensitivity DNA kit. dsDNA concentrations were determined using the Qubit High Sensitivity kit and twelve random libraries were pooled in equimolar amounts. Sequencing of the degradome libraries was performed by the McGill University and Genome Quebec Innovation Centre. The clustering was done on an Illumina cBot and the sequencing was performed on a HiSeq 2000 for 50 cycles. A *phi*X library was used as control at 10% level. bcl2fastq v1.8.4 (Illumina) was used to demultiplex the sample reads and generate the fastq files.

Raw reads were quality-checked and adapter sequences were removed using a custom Perl script. Reads ranging from 19 to 21 nt were extracted using Geneious (Biomatters Ltd., Auckland, NZ) and were converted to distinct tags using Tally [53]. The distinct tags were mapped to the coding sequences of wheat (IWGSC RefSeq v1.0, 2018) using sPARTA [54]. The previously annotated miRNA sequences were queried for target prediction using the sPARTA’s miRferno target prediction module. miRNA targets and their abundance were extracted and target plots were constructed using the R package ggplot2. miRNA targets with a threshold *P* value < 0.05 and read abundance > 4 were retained for further analysis.

### Prediction of heat stress associated regulatory networks

Degradome validated miRNA targets were extracted and translated to query the Search Tool for the Retrieval of Interacting Genes/Proteins (STRING version 10.5) database [55]. Orthologous proteins matching rice (*Oryza sativa* ssp. indica L.) with identity > 50% were used to associate coexpression and interactome data. A high confidence of 0.7 was used to construct the regulatory networks.

### Data availability and accession numbers

The smallRNA and degradome (PARE) data were submitted to the NCBI Gene Expression Omnibus under accession number GSE113358.

## Results

### Small RNA profile and annotation of microRNAs

A total of 24 sRNA libraries, obtained from leaf tissues harvested from four biological replicates representing control and heat stressed plants sampled at 0, 1 and 4 DAT were sequenced. After quality check and removal of low quality reads, a total of 55.2 M 18–24 nt-long reads corresponding to 18.7 M distinct tags were retained for miRNA annotation (Additional file 1). The number of high quality raw reads and distinct tags was comparable regardless of the treatment and sampling time (Additional file 2: Figure S1a,b). A size distribution analysis of raw reads showed that the 21- and 24-nt size classes exceeded other size classes (Additional file 2: Figure S1c) and the proportion of distinct tags was skewed towards the 24-nt size class (Additional file 2: Figure S1d).

**Fig. 1:**
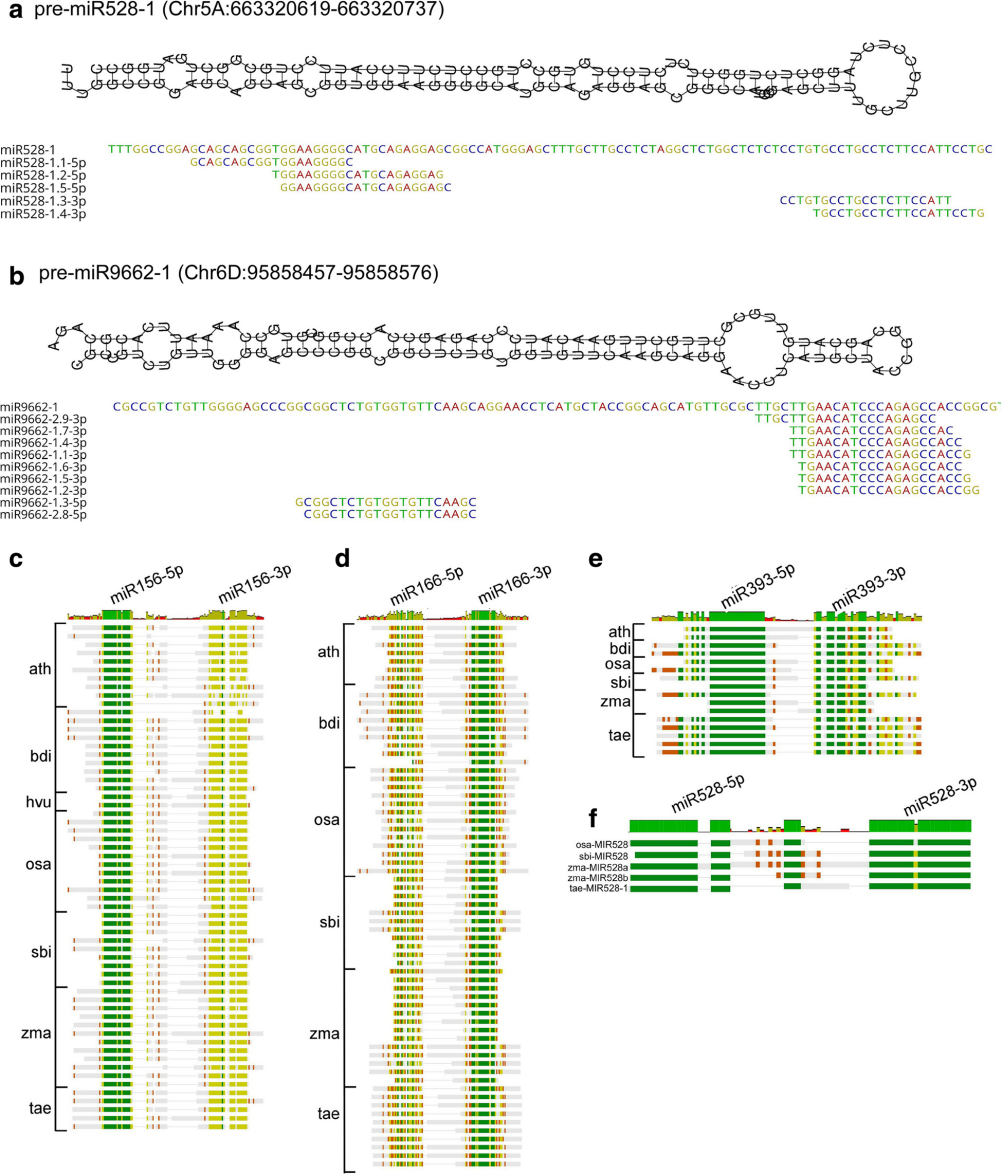
Foldback structures and sequence conservation among miRNA families. Schematic drawings of the foldback structure of pre-miRNA528–1 (**a**) and pre-miRNA9662–1 (**b**) and the multiple alignments of their encoded isomiRs. Alignments of miR156 (**c**), miR166 (**d**), miR393 (**e**) and miR528 (**f**) precursors from *Arabidopsis thaliana* (ath), *Brachypodium distachyon* (bdi), *Oryza sativa* (osa)*, Sorghum bicolor* (sbi)*, Hordeum vulgare* (hvu), *Zea mays* (zma) and *Triticum aestivum* (tae; our study). Identity is color-coded as follows: dark green (100%), light green (80–100%), orange (60–80%) and grey (< 60%)

The adapter trimmed, high quality reads were filtered and annotated using the in-house pipeline described in the methods to identify a total of 202 mature miRNAs belonging to 37 miRNA families (Additional file 1, Additional file 3: Figure S2a). Evidence for complementary miRNA star (miRNA*) sequence on the other arm of the precursor with 2-bp overhang was found for 104 mature miRNAs. The majority (161/202) matched more than one pre-miRNA coding loci across all sub-genomes (Additional file 4). Here, miRNAs derived from a single precursor are termed isomiRs. For example, all five isomiRs of miR528 were processed from the single pre-miRNA miR538–1 transcribed from locus Chr5A:663320619–663,320,737 (Fig. 1a). Similarly, all nine 19–21 nt-long isomiRs of miR9662 were processed from pre-miR9662–1 aligning to Chr6D:95858457–95,858,576 (Fig. 1b). IsomiRs processed from these two pre-miRNAs differed among themselves by minor shifts of one to three nucleotides (Additional file 4; Fig. 1a,b). A search for orthologous pre-miR528 in miRBase (release 22), identified a single miRNA coding locus in rice (*Oryza sativa*) and sorghum (*Sorghum bicolor*) while maize (*Zea mays*) carried two loci on chromosomes 1 and 9, respectively. A similar search for pre-miR9662 failed to identify orthologous precursors.

Multiple sequence alignment of orthologous precursors revealed a high degree of sequence conservation primarily in the miRNA/miRNA* region of the foldback structure (Fig. 1c-f). The position of sequence conservation varied among miRNA families in the foldback structures. Orthologous precursors of miR156 were highly conserved at the 5p arm of the foldback structure and all mature miRNAs were derived from the same (Additional file 4; Fig. 1c). Precursors of miRNA166 were highly conserved at the 3p arm but not so at the 5p arm. Of the 23 mature miR166, 20 were derived from the 3p arm. The less diverse and lineage specific families such as miR393 and miR528 were conserved at both 5p and 3p arms of the precursor (Fig. 1e,f).

Among the 37 miRNA families, 24 families comprised more than one mature miRNAs (Additional file 3: Figure S2a). Families miR1135 and miR166 were the most diverse with 27 and 23 mature miRNAs, respectively. miR1137 comprised only 21 and 24-nt size classes, while 58% (7/12) of miR1118 were 24-nt long (Additional file 3: Figure S2b). The conserved 21-nt size class represented 57% of the miRNA distinct tags corresponding to 91% of the raw reads (Additional file 5).

### Relative abundance and differential expression of heat stress regulated miRNAs

Of the 37 families, 29 were weakly expressed representing less than 1% of the reads (Additional file 6: Figure S4a). Despite the presence of 27 distinct miRNAs in family miR1135, this family accounted for only 0.5% of the reads. In contrast, family miR166 was the most abundant totalling 79% of the reads, including six members with more than 1000 RPM in all libraries and the remaining 17 ranging from 10 to 1000 RPM (Additional files 7 and 8). miR166–4.1-3p was the most abundant with an average read count of 414,544 RPM. Overall, ten miRNA families had read counts exceeding 1000 RPM which globally represented more than 90% of all reads (Additional file 6: Figure S4b).

**Fig. 2:**
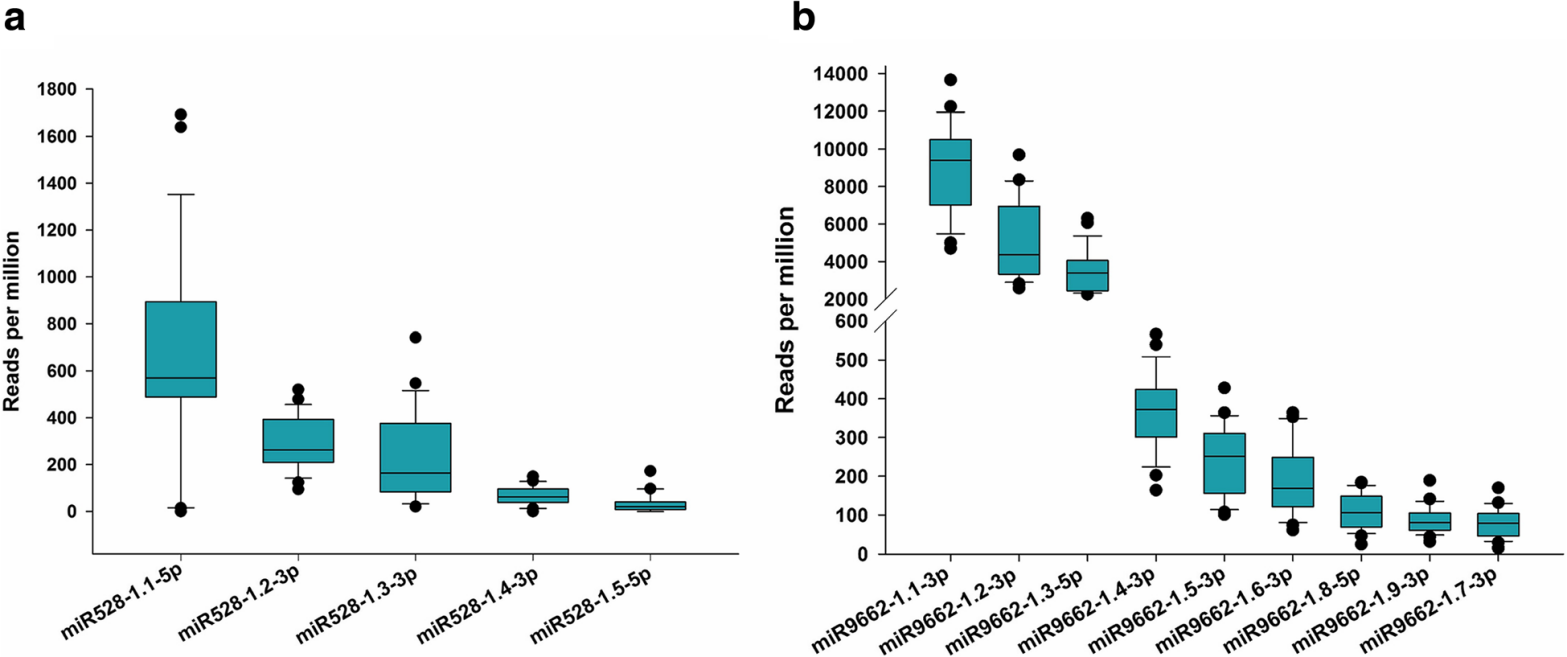
Box plots of the relative abundance of isomiRs belonging to miRNA family (**a**) miR528 and (**b**) miR9662. The median is shown by a horizontal black line. Boxes represent the 25th and 75th percentiles while error bars indicate the 10th and 90th percentiles

Difference in accumulation was also observed among miRNA processed from the same precursor, such as the case with miR528 and miR9662 where isomiRs varied from 33 to 660 RPM and 14 to 13,658 RPM, respectively (Additional file 8; Fig. 2a,b). Analysis of differences in accumulation of mature miRNAs and their complementary miRNAs* showed the abundance of mature miR9662–1.4-3p to be considerably lower (8738 RPM) than its complementary star miR9662–1.3-5p (82,966 RPM) (Additional file 9: Figure S6a). Inversely, although not nearly as pronounced, the abundance of mature miR9662–1.7-3p was lower (1928 RPM) than its complementary star miR9662–1.8-5p (2591 RPM) (Additional file 9: Figure S6b).

**Fig. 3:**
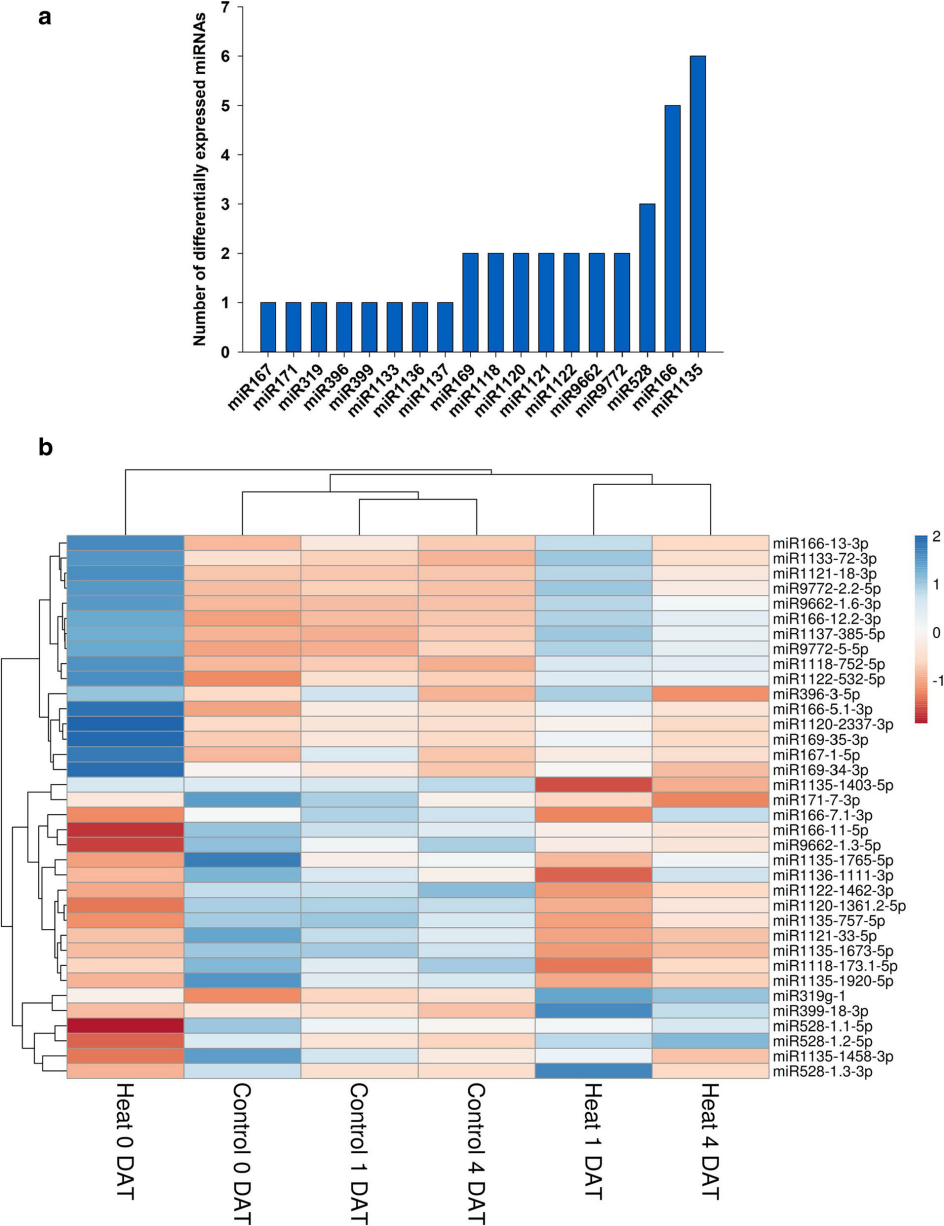
Differential expression of miRNAs following heat stress. (**a**) Distribution of the 36 miRNAs differentially expressed in wheat leaf tissue after heat stress into 18 families (FDR < 0.05). (**b**) Heat map of the 36 differentially expressed miRNAs in response to heat stress. The blue color represents upregulation and red is for downregulation. The dendrogram represents the hierarchical clustering of the control and heat treatments at the three sampling time points of 0, 1 and 4 days after treatment (DAT)

An MDS plot constructed using the normalized read counts showed the relative similarities among the biological replicates (Additional file 10: Figure. S7). Of the 202 mature miRNAs, 36 belonging to 18 families were differentially expressed upon heat stress (Additional file 11; Fig. 3a). Cluster dendrogram of differentially expressed miRNA revealed a temporal expression pattern where the heat-stress induced alterations in miRNA levels were more pronounced immediately at the end of the heat stress (0 DAT; Fig. 3b). For the majority of the differentially expressed miRNAs, fold change expression differences were smaller during the recovery phase at 1 and 4 DAT compared to 0 DAT (Fig. 3b). Of the 36 differentially expressed miRNAs, 18 were downregulated immediately after heat stress (0 DAT; Additional file 11). Hierarchical clustering of differentially expressed miRNAs showed that miR528 and miR1135 were downregulated after heat stress (Fig. 3b). In contrast, members of miR169 and miR9772 were upregulated by the end of the 5-day heat stress period (Fig. 3b; Additional file 11). Levels of miR1121–33-5p were reduced in response to heat stress, but unlike others, they did not return to the same level as the control during the recovery period (1–4 DAT; Additional file 11). Members of miR166, miR1118, miR1120, miR1121 and miR1122 were also differentially regulated in response to heat stress (Fig. 3a,b). Comparison between heat 0 and 4 DAT identified nine differentially expressed miRNAs: miR528–1.1-5p, miR528–1.3-3p, miR166–11-5p, miR528–1.2-5p, miR399–18-3p, miR166–13-3p, miR167–1-5p, miR1120–2337-3p, and miR166–5.1-3p (Fig. 3b). Comparison between control 0 and 4 DAT did not reveal any differentially expressed miRNA.

### Identification of cleaved miRNA targets through sequencing of the degradome

**Fig. 4:**
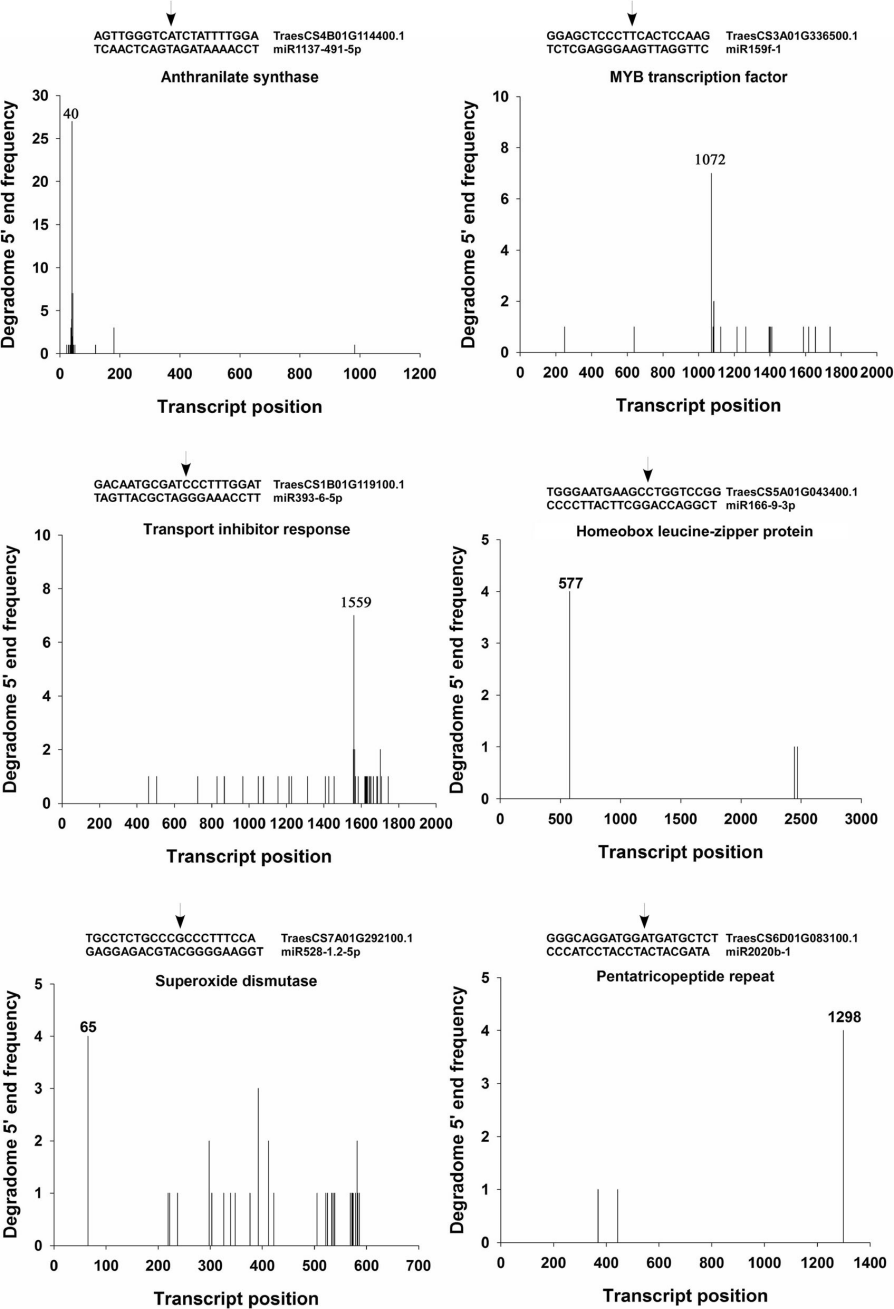
Identification of cleaved miRNA targets. The target plots (t-plots) show read abundance aligned to the indicated transcript. The arrows above the miRNA-transcript alignment indicate the miRNA-directed cleavage site

Sequencing of the 24 degradome libraries yielded a total of 404 M reads. After quality check and adapter trimming, 370 M 19–21 nt-long reads were analyzed. A total 84 miRNAs were identified to target 589 transcripts corresponding to 206 protein coding loci (*P* value < 0.05; PARE abundance > 4; Additional file 12) thus validating several conserved miRNA targets (Table 1). IsomiRs of miR156 and miR159 targeted squamosa promoter-binding-like proteins and a MYB transcription factor, respectively (Fig. 4). Homeobox leucine-zipper proteins were targeted by no less than 20 miRNAs of family miR166. Similarly, stress responsive mitochondrial transcription termination factor-like proteins were targeted by three isomiRs of miR9662.

**Table 1:** Selected miRNA targets validated through degradome sequencing. Conserved miRNA and their targets are italicized

miRNA	Target of miRNA	Functional pathways
*miR156–1.1-5p*	*Squamosa promoter-binding-like protein*	Stress signalling, regulation of juvenile state [88]
*miR156–1.2-5p*		
*miR159f-1*	*MYB transcription factor*	Anther development and male sterility [33]
*miR166–12.1-3p*	*Homeobox leucine-zipper protein*	Hormone signalling, leaf morphogenesis and development [89]
miR166–12.2-3p*		
miR166–13-3p*		
*miR166–1-3p*		
*miR166–3.1-3p*		
*miR166–3.2-3p*		
*miR166–4.1-3p*		
*miR166–4.2-3p*		
*miR166–4-3p*		
miR166–5.1-3p*		
*miR166–5.2-3p*		
*miR166–6-3p*		
miR166–7.1-3p*		
*miR166–7.2-3p*		
*miR166–8.1-3p*		
*miR166–8.2-3p*		
*miR166–8.3-3p*		
*miR166–8.5-3p*		
*miR166–8.6-3p*		
*miR166–9-3p*		
*miR167–14-3p*	*Auxin response factor*	Auxin signalling [90, 91]
miR167–1-5p*		
*miR167–18-5p*		
*miR167–2-5p*		
*miR167–35-5p*		
*miR169–51-3p*	*Nuclear transcription factor Y subunit*	ABA signalling and drought stress signalling [92]
*miR169l-1*		
*miR171–2-3p*	*Scarecrow transcription factor family protein*	Floral development drought stress signalling [92]
*miR393–2-5p*	*Transport inhibitor response 1*	Auxin signalling [93]
*miR393–4-5p*		
*miR393–6-5p*		
*miR395–18-3p*	*ATP sulfurylase (Sulfate adenylyltransferase)*	Sulfate transport [92]
*miR395–41-3p*		
*miR398–1.2-3p*	*Superoxide dismutase [Cu-Zn]*	Antioxidant activity [94]
miR528–1.2-5p*, ^*†*^	*Superoxide dismutase [Cu-Zn], L-ascorbate oxidase*	Antioxidant activity [78]
miR2020b-1	Pentatricopeptide repeat-containing protein	RNA metabolism and organ development [82, 83]
miR9662–1.1-3p	Mitochondrial transcription termination factor-like	Regulation of transcription
miR9662–1.2-3p		
miR9662–1.4-3p		
miR9662–1.5-3p		
miR9662–1.6-3p*		
miR9662–1.7-3p		
miR9662–2.9-3p		
miR9670–2.1-5p	Mitochondrial transcription termination factor-like	Regulation of transcription [85]
miR9772–1-5p^‡^	F-box proteins	[22, 79]
miR9772–2.1-5p^‡^		
miR9772–2.2-5p*,^‡^		
miR9772–4-5p^‡^		
miR9772–5-5p*,^‡^		
miR9772–6-5p^‡^		

*significant differential expression at any time points after heat stress (FDR < 0.05);^†^ indicates monocot specific miRNAs, ^‡^ indicated *Triticum* specific miRNAs

Transcripts associated with superoxide dismutase activity were targeted by both miR398–1.b-3p and miR528–1.b-5p. Kinases and transcripts involved in ubiquitin-mediated proteolysis were regulated by several miRNAs from multiple families including miR1118, miR1120, miR1135 and miR1136. F-box family proteins were targeted by isomiRs of miR1133, miR1135, miR1136 and miR9772. Anthranilate synthase was targeted by isomiRs of family miR1136, miR1137 and miR1439 (Table 1).

### Prediction of heat stress associated regulatory networks

**Fig. 5:**
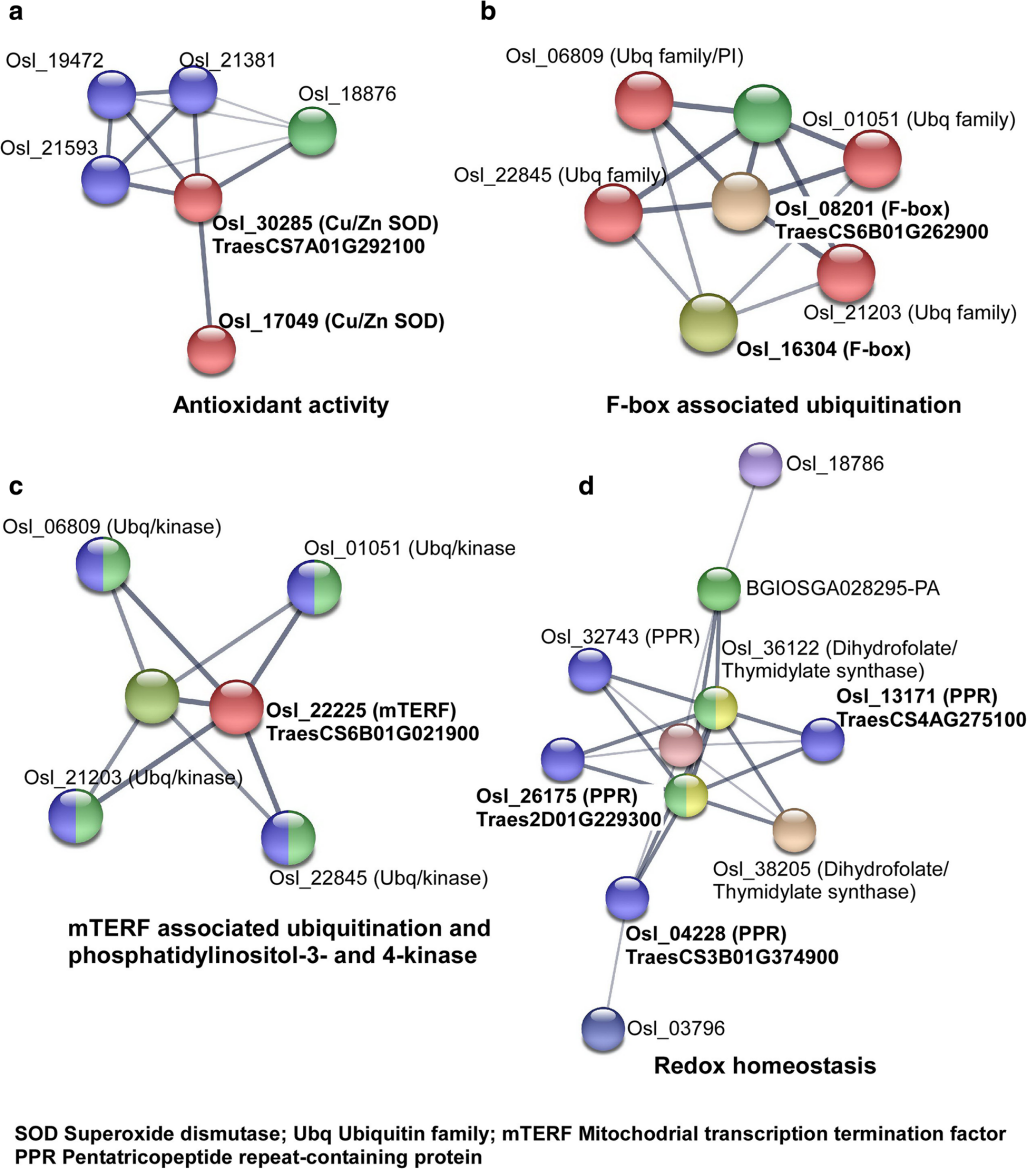
STRING network view of known and predicted interaction partners. Degradome validated miRNA targets were used as query to search orthologous proteins in rice (*Oryza sativa*). The confidence view screen shows the known and predicted protein-protein interactions where stronger associations are represented by thicker lines. The bold font indicate the queried protein

Degradome validated miRNA targets queried to STRING DB predominantly associated with redox homeostasis, antioxidant activity and ubiquitination (Fig. 5). Cu-Zn Superoxide dismutases targeted by miR528 and miR398 coexpressed with other Fe/Zn Superoxide dismutases. Cellular and redox homeostasis related network were also observed with pentatricopeptide repeat-containing protein (PPR) involving dihydrofolate reductase/thymidylate synthase. A prominent interaction cluster was observed for F-box associated with ubiquitin family of proteins. Query of mitochondrial transcription termination factor (mTERF) resulted in a similar cluster associating both ubiquitin family and phosphatidylinositol 3- and 4-kinase domain carrying proteins. Predicted interacting proteins and their annotation are listed (Additional file 13).

## Discussion

Understanding the ability of plants to sense and respond to abiotic stresses such as heat has implications in plant breeding. In recent years, the prominent role of miRNA-guided post-transcriptional gene regulation has been uncovered in plant development and stress responses [reviewed by [31, 56, 57] ]. In this study, we report on the regulatory role of miRNAs in response to heat stress by analyzing an interlaced data set of sRNA and degradome sequences. With stringent annotation guidelines [43], we identified 202 mature miRNAs, of which, 36 were differentially altered with temporal expression profile upon heat stress. Through degradome analysis, we identified 84 miRNA targeting 206 protein coding loci. As reported for other plant species, a high degree of conservation was observed in miR156 regulating squamosa promoter-binding-like protein, miR159 regulating MYB transcription factor, miR166 regulating homeobox leucine-zipper protein and miR398 regulating superoxide dismutase [22, 31]. In addition to the typical heat stress response target transcripts of superoxide dismutases, peroxidases and an array of transcription factors, we also identified miRNA targets for organelle specific transcripts such as pentatricopeptide repeat-containing proteins and mitochondrial transcription termination factor-like proteins through miRNA-guided cleavage; a phenomenon that has not previously been reported in monocots.

Polyploid crops such as wheat have evolutionary advantages in development and adaptation [reviewed by [58] ]. Polyploid genomes better tolerate gene loss or silencing compared to their diploid counterparts because of their innate functional redundancy [59]. Hybridization and whole genome duplication have been demonstrated to result in expansion of miRNA families and enhance stress tolerance [60, 61]. Here, isomiRs of heat stress responsive miR528 and miR9662 were processed from single pre-miRNAs to regulate ROS and mitochondrial transcription termination factor-like proteins, respectively. The absence of the homoeologous coding loci may be from miRNA gene loss on the homoeologues or, alternatively, from selective gain in allohexaploid wheat. Such cases were however the exception rather than the norm considering that 161 miRNAs aligned to more than one pre-miRNA across all sub-genomes with many being highly redundant. For instance, miR1136–235-3p, targeting the 30S ribosomal protein S3, aligned to 1725 pre-miRNA coding loci across all sub-genomes. Previous studies on allohexaploid wheat and their progenitors have shown non-additive expression of miRNAs to regulate growth and adaptability [62]. Allohexaploid wheat and their progenitors are known to carry sub-genome donor- specific miRNA profiles [63].

Plant pre-miRNAs are variable in size and foldback structure compared to their animal counterparts [23]. The presence of one to three nucleotide bulges, mismatches and large loops in the foldback structure alter DCL recognition and processing resulting in new miRNA variants or isomiRs [24, 64]. In our study, all five isomiRs of miR528 and nine isomiRs of miR9662 were processed from pre-miRNA miR528–1 and miR9662–1, respectively. Despite being processed from the same pre-miRNAs, these isomiRs were not produced in equimolar amounts and only a subset of them was differentially expressed after heat stress. Mature miRNA accumulation is dictated by the rate of transcription, processing and their 3′ end modifications, the latter being typically methylated to be protected [65] from 3′ terminal uridination and subsequent degradation [66]. Previous studies have also shown isomiRs accumulation to vary across tissue types and in response to external stimuli such as stresses [67, 68]. Hence, it is imperative to quantify isomiRs because they differ in function and their expression from a single locus is controlled at the post-transcriptional level.

miRNA genes are transcribed independently based on their own promoter activity, a likely explanation for the differential accumulation of miRNAs of the same family. Although, members of miR166 were transcribed by only 16 miRNA coding loci, they accounted for more than 70% of the reads. IsomiRs of miR166 were also predominant in leaf tissue of durum wheat [69] and soybean [70]. Differences were also observed in accumulation of mature miRNA or guide strand and the complementary miRNA originating from the opposite arm, often called miRNA-star(*) (Additional file 9). The most abundant mature miRNAs were considered the dominant and functional products that regulate PTGS, whereas the miRNA* were considered minor products of the duplex processing [71]. However, miRNA* should not be discounted because it also has the ability to regulate gene expression [72]. The abundance of miRNA* miR9662–1.3-5p and miR9662–1.8-5p was higher than their corresponding miRNAs and both were differentially altered in response to heat stress, indicating a possible functional role in wheat (Additional file 11). However, despite their differential expression, we did not observe accumulation of their cleaved mRNA products in the degradome dataset. Nevertheless, three isomiRs processed from the 3p arm of pre-miR9662–1 were identified to target mitochondrial transcription termination factor-like protein encoding genes, lending credence to their functional role. Studies on Arabidopsis have shown that accurate processing and miRNA guide strand selection are regulated by HYL and CPL1 phosphatases [73]. The loading of miRNA to specific AGO is directed by the 5′ terminal nucleotides and mismatches in the duplex structure [74]. Consequently, it is possible that miRNAs processed from 3p and 5p arms can be preferentially selected to be loaded into different AGO complexes to regulate different transcripts in a tissue and stress specific manner.

The temporal expression profile of miRNAs in wheat cv Chinese Spring after heat stress is similar to our previous report on wheat cv Glenlea [37]. However, the relative accumulation of miRNAs varied between the two cultivars. For example, miR398 and miR169 were abundant in cv Glenlea whereas miR1135 and miR166 were the most abundant in cv Chinese Spring. These variations can occur as a consequence of shear genetic and regulatory differences among cultivars as reported in cowpea [39], cotton [38] and rice [75]. sRNA and degradome sequencing confirmed that few miRNA families and their targets were generally conserved across many plant species. miRNA families 156, 159, 166, 393 and 398 targeting squamosa promoter-binding-like proteins, MYB transcription factors, homeobox leucine-zipper proteins, transport inhibitor response proteins and superoxide dismutases, respectively, are highly conserved across dicots and monocots [31, 76]. In addition to conserved miRNA families and their targets, monocot specific miR528 was differentially altered after heat stress to regulate antioxidant activity. Heat stress induced H_2_O_2_ has been shown to function as a key signalling molecule to regulate the expression of heat shock proteins [7, 77]. The transient downregulation of miR528 and validation of the guided cleavage of the superoxide dismutase target through degradome sequencing exemplify a miRNA-directed stress response. A recent study has also shown that suppression of miR528 enhances antiviral defense in rice [78]. *Triticum*-specific miR9772 was validated to target F-box family proteins, implicating the regulating ubiquitin-mediated proteasome degradation. In Arabidopsis, F-box family proteins are regulated by miR393 and miR394 to promote ubiquitination and proteasome degradation [22]. Overexpression of wheat F-box protein TaFBA1 confers oxidative and drought stress tolerance with enhanced antioxidant activity [79]. Anthranilate synthase, mediating the biosynthesis of chorismate to tryptophan, was targeted by miR1137–229-3p. In plants, tryptophan serves as a precursor for the synthesis of phytohormone auxins, phytoallexins, glucosinolates and alkaloids to control an array of developmental processes and to confer biotic and abiotic stress tolerances [80]. Heat stress associated regulator network revealed distinct clusters linking biosynthesis of tryptophan, phytohormones, antioxidant activity and ubiquitination. Although, six transport inhibitor response proteins were identified as targets of miR393, only one was differentially expressed with the expected inverse correlation. Sub-genome specific transcriptional level gene regulation and relative rates of biogenesis and decay of miRNA and their targets are possible [81].

miRNA-guided gene expression was also observed among organelle specific regulatory proteins. For instance, miR2020 targeted chloroplast and mitochondria localized a pentatricopeptide repeat-containing protein (PRP) that facilitates processing, splicing, editing, stability and translation of RNAs [82]. In rice, loss of pentatricopeptide repeat-containing proteins (WSL1) led to enhanced sensitivity to salinity, sugar and abscisic acid [83]. Similarly, mitochondrial transcription termination factor-like protein (mTERF) was targeted by miR9662. PRP and mTEFR carry tandem degenerate helical repeats and direct RNA splicing [84]. Studies on Arabidopsis and maize have shown that mTERF regulates responses to high light [85], heat stress [86] and salinity [87].

## Conclusion

The interlaced data set generated in this study identified and validated heat stress regulated miRNAs and their target genes associated with thermotolerance. We showed that miRNAs were highly altered immediately after heat stress to regulate thermotolerance but expression levels largely returned to control levels during the 1–4 day period following the end of the stress. Degradome sequencing revealed that the target genes of miRNA156, miR159, miR166 and miR398 are conserved among wheat, other cereal crops and dicot plants. Chloroplast and mitochondria localized pentatricopeptide repeat-containing and mitochondrial transcription termination factor-like proteins were shown to be regulated through miRNA-guided cleavage, a phenomenon that has not been reported to date in monocots. A thorough understanding of miRNA-mediated epigenetic changes constitutes an additional source of variation that can be capitalized upon for developing new breeding strategies targeting expression rather than structural changes.

## Additional files

Additional file 1: **Table S1** Sequencing and processing summary of the 24 small RNA libraries. (XLSX 19 kb)

Additional file 2: **Figure S1.** Overview of small RNA profile. **a** Distribution of raw reads by treatment and sampling time point. **b** Distribution of distinct tags by treatment and sampling time point. The median is shown by a horizontal black line. Boxes represent the 25th and 75th percentiles while error bars indicate the 10th and 90th percentiles. **c** Size distribution of raw reads after size selection. **d** Size distribution of distinct tags prior to removal of other non-coding RNA and chloroplast sequences. (TIF 895 kb)

Additional file 3: **Figure S2.** miRNAs families. (a) Number of isomiRs in the 37 miRNA families identified in heat stress and control wheat leaves. (b) Relative abundance of size classes within the same conserved 37 miRNA families. (TIF 473 kb)

Additional file 4: **Table S2.** Conserved miRNA families, precursor sequence, structure, minimum free energy and star sequence information. (XLSX 54 kb)

Additional file :5 **Figure S3.** Size distribution of miRNA families represented as percentage of raw reads and distinct tags. (TIF 151 kb)

Additional file 6: **Figure S4.** Relative abundance of miRNAs families expressed in heat stress and control wheat leaves. (a) Normalized raw read percentages of miRNA families. (b) Relative abundance of the normalized read counts based on the levels of expression in reads per million (RPM). (TIF 490 kb)

Additional file 7: **Figure S5.** Box plots of the relative abundance of mature miRNAs belonging to family miR166. The median is shown by a horizontal black line. Boxes represent the 25th and 75th percentiles while error bars indicate the 10th and 90th percentiles. (TIF 271 kb)

Additional file 8: **Table S3.** Normalized read counts of annotated miRNAs for each of 24 small RNA libraries corresponding to two treatments (control and heat), four replicates and three sampling time points (0, 1 and 4 days after treatment). (XLSX 71 kb)

Additional file 9: **Figure S6.** Abundance of miRNAs cleaved from the 5p and 3p ends of pre-miRNA9662. (a) miR9662–1.3-5p/miR9662–1.4-3p and (b) miR9662–1.8-5p/miR9662–1.7-3p. (TIF 218 kb)

Additional file 10: **Figure S7.** MDS plot displaying the relative similarities among the biological replicates. (TIF 148 kb)

Additional file 11: **Table S4.** List of miRNA differentially expressed after heat stress. (XLSX 17 kb)

Additional file 12: **Table S5.** List of miRNA targets identified by degradome sequencing. (XLSX 598 kb)

Additional file 13: **Table S6.** List of predicted interaction partners for degradome validated miRNA targets identified in rice (*Oryza sativa*) using STRING DB. (XLSX 15 kb)

## Data Availability

The smallRNA and degradome (PARE) data were submitted to the NCBI Gene Expression Omnibus under accession number GSE113358.

## Abbreviations

DAT: days after treatment
miRNA: microRNA
nt: nucleotide
PARE: Parallel Analysis of RNA Ends
RPM: reads per million
