# Supplementary figures and images for: MicroRNA-guided regulation of heat stress response in wheat
Source: BMC Genomics. 2019 Jun 13;20:488. doi: 10.1186/s12864-019-5799-6 (PMC6567507; doi:10.1186/s12864-019-5799-6)

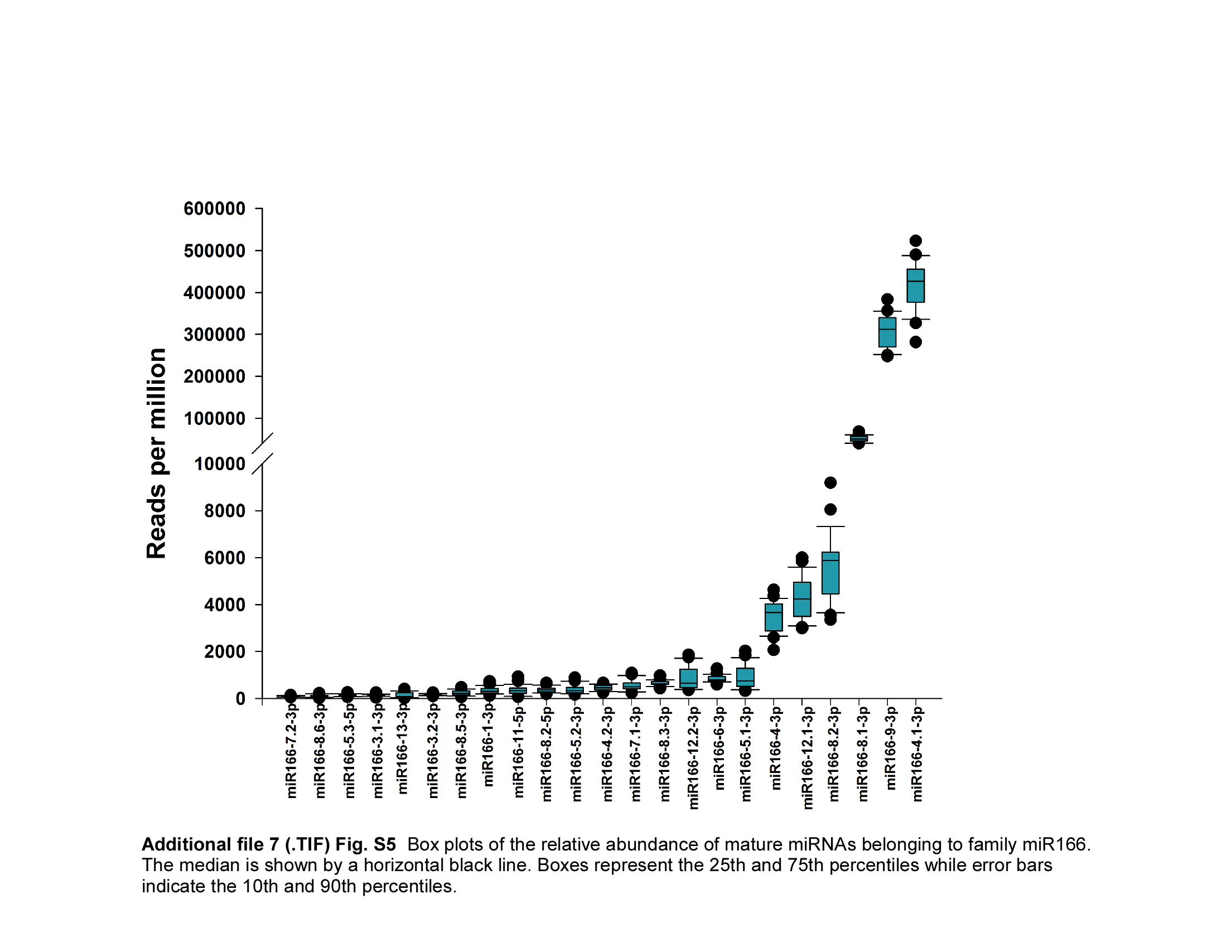

Supplement: Supplementary file 7 — Figure S5. Box plots of the relative abundance of mature miRNAs belonging to family miR166. The median is shown by a horizontal black line. Boxes represent the 25th and 75th percentiles while error bars indicate the 10th and 90th percentiles. (TIF 271 kb) [file 12864_2019_5799_MOESM7_ESM.tif]

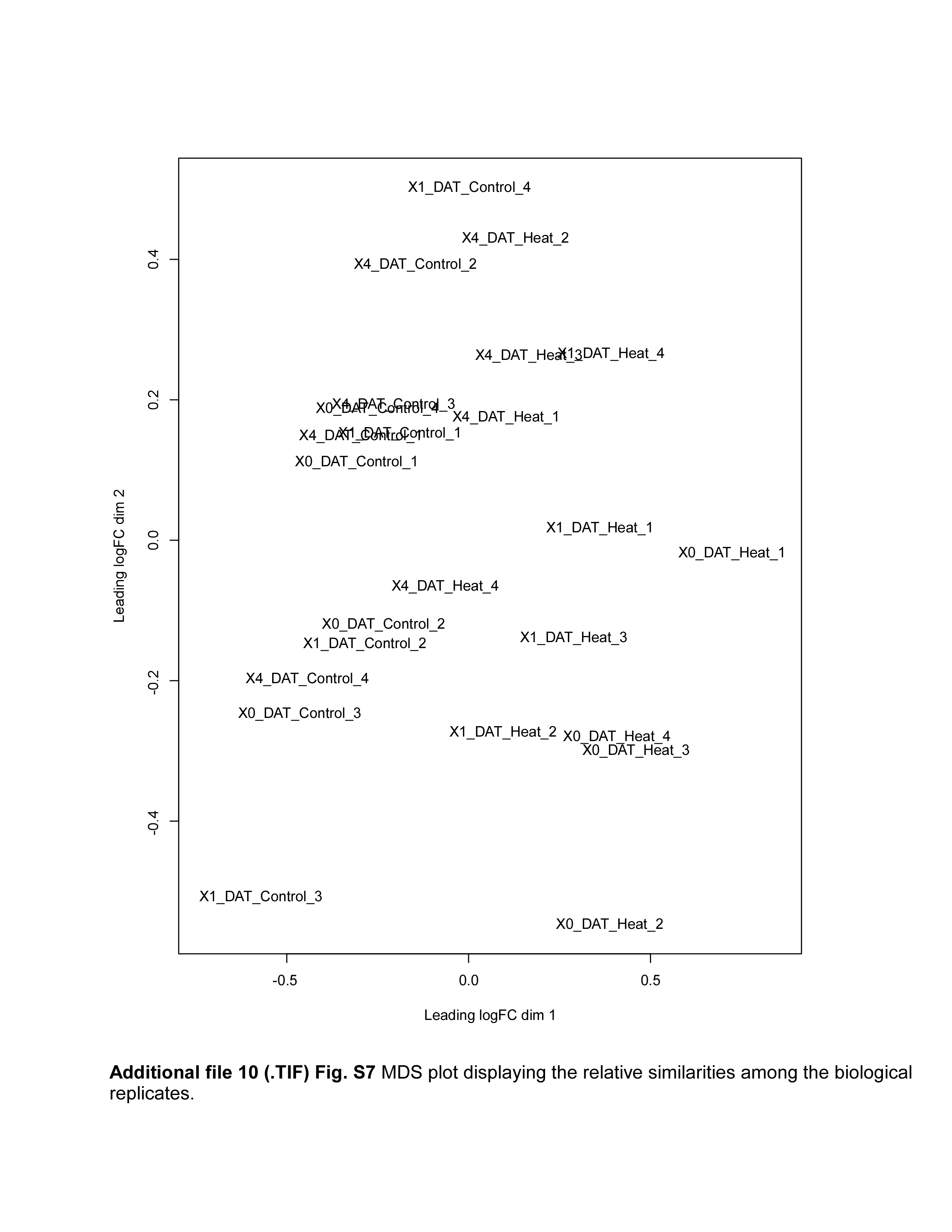

Supplement: Supplementary file 10 — Figure S7. MDS plot displaying the relative similarities among the biological replicates. (TIF 148 kb) [file 12864_2019_5799_MOESM10_ESM.tif]
